# Catalytic Bioscavengers Against Toxic Esters, an Alternative Approach for Prophylaxis and Treatments of Poisonings

**Published:** 2009-04

**Authors:** Patrick Masson, Daniel Rochu

**Affiliations:** 1 Centre de Recherches du Service de Santé des Armées, Toxicology department, La Tronche, France;; 2 Institute of Structure Biology, Molecular Biophysics Laboratory, Grenoble, France;; 3 Bundeswehr Institute of Toxicology and Pharmacology, Munich, Germany

## Abstract

Bioscavengers are biopharmaceuticals that specifically react with toxicants. Thus, enzymes reacting with poisonous esters can be used as bioscavengers for neutralization of toxic molecules before they reach physiological targets. Parenteral administration of bioscavengers is, therefore, intended for prophylaxis or pre-treatments, emergency and post-exposure treatments of intoxications. These enzymes can also be used for application on skin, mucosa and wounds as active components of topical skin protectants and decontamination solutions.

Human butyrylcholinesterase is the first stoichiometric bioscavenger for safe and efficient prophylaxis of organophosphate poisoning. However, huge amounts of a costly enzyme are needed for protection. Thus, the bioscavenger approach will be greatly improved by the use of catalytic bioscavengers. Catalytic bioscavengers are enzymes capable of degrading toxic esters with a turnover.

Suitable catalytic bioscavengers are engineered mutants of human enzymes. Efficient mutants of human butyrylcholinesterase have been made that hydrolyze cocaine at a high rate. Mutants of human cholinesterases capable of hydrolyzing OPs have been made, but so far their activity is too low to be of medical interest. Human paraoxonase a promiscuous plasma enzyme is certainly the most promising phosphotriesterase. However, its biotechnology is still in its infancy. Other enzymes and proteins from blood and organs, and secondary biological targets of OPs and carbamates are potential bioscavengers, in particular serum albumin that reacts with OPs and self-reactivates. Lastly, non-human enzymes, phosphotriesterases and oxidases from various bacterial and eukaryotic sources could be used for external use against OP poisoning and for internal use after modifications for immunological compatibility.

## 1. The bioscavenger concept

Enzyme systems located in skin, blood organs are involved in natural defences against endogenous and exogenous poisons. Detoxification processes occur through different types of reactions, including oxidation, hydrolysis, and conjugation. The role of liver, lung and kidney enzymes, cytochromes P450 (Brown et al., 2008), oxidases, transferases (Miners et al., 2006), amido-carboxylesterases (Redinbo and Potter, 2005; Potter and Wadkins, 2006; Satoh and Hosokawa, 2006) in the metabolism of drugs and xenobiotics is well known. The importance of plasma esterases in the inactivation of numerous toxicants has been recognized, too. Lastly, there is growing evidence that catalytic antibodies play also an efficient role in scavenging deleterious molecules and radicals (Belogurov et al., 2009). These multiple enzymes constitute cellular and circulating barriers that protect physiological machineries and systems against specific toxicants. Here we will examine endogenous and exogenous enzymes that react with poisonous carboxylic-, organophosphoryl- and carbamyl-esters. These enzymes act either as stoichiometric bioscavengers or catalytic bioscavengers. Catalytic bioscavengers are biocatalysts capable of degrading poisonous compounds with a turnover. Enzymes that are potential catalytic bioscavengers will be reviewed. 

**Fig. 1.**
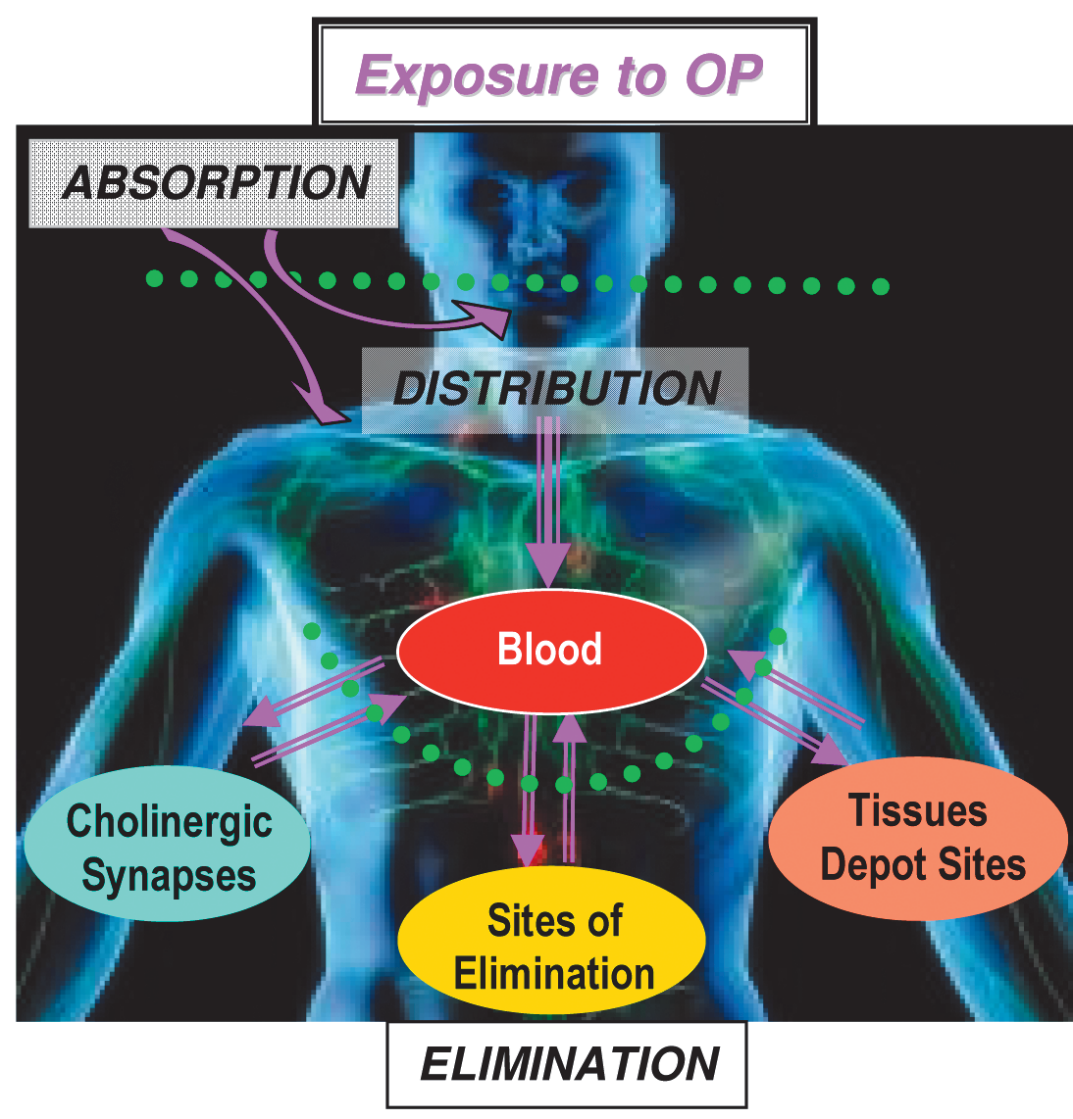
Biological fate of organophosphorus compounds in humans. Routes of penetration of OPs are absorption through the skin, eyes, and/or respiratory tract (nerve agents, pesticides), or ingestion (self-poisoning). OP molecules distribute from the blood compartment into tissues, including depot sites, biophase (physiological targets), and sites of elimination (liver and kidneys). ChEs of cholinergic synapses are the primary targets; their inhibition is responsible for the acute toxicity of OPs; reaction with secondary targets (carboxylesterases, serine-amidases, peptidases and other serine/tyrosine proteins) may be responsible for the non-cholinergic sub-lethal effects of OPs and chronic toxicity at low-dose exposure (Casida and Quistad, 2004; Costa, 2006).

After a short survey of catalytic bioscavengers against cocaine, we will focus on biocatalysts to be used for prophylaxis and treatment of organophosphate poisoning. Indeed, OP poisoning is a major public-health problem. OP self-poisoning is responsible for 200,000 deaths a year in the world (Eddelston et al., 2008). In addition, though 185 nations have joined the Chemical Weapons Convention (CWC), nerve agents and other organophosphorus compounds still represent military and terrorist threats. Significant progress has been made in the past twenty years in countermeasures of OP poisoning (Aas, 2003; Albuquerque et al., 2006; Wetherell et al., 2007; Eyer et al., 2007; Thiermann et al., 2007). However, classical pharmacological approaches have reached their optimum limit. Toxicity of OPs can be countered by reducing skin absorption and lowering OP concentration in the blood compartment, thus preventing the transfer of OP molecules towards physiological targets (Fig. 1). Neutralization of OPs has proved to be possible by using stoichiometric traps, first generation bioscavengers. The catalytic bioscavenger concept, second generation bioscavengers, is based on the idea of continuously trapping and degrading OPs in the blood stream before OP molecules reach their central and peripheral neuronal and neuromuscular targets.

## 2. Detoxification of (-)cocaine

Unlike the plasma of most mammalians, there is no carboxylesterase in human plasma (Li et al., 2005). However, two enzymes are capable of degrading esters in the blood stream. Plasma paraoxonase (PON1; EC 3.1.8.1) displays an arylesterase activity, and butyrylcholinesterase (BChE; EC 3.1.1.8) — that has broad esterase specificity — plays a role in processing, catabolism and or detoxification of numerous poisonous esters: for instance, human BChE hydrolyzes ester-containing therapeutic and/or addictive drugs such as succinylcholine and its long-chain derivatives (Grigoryan et al., 2008), aspirin, irinotecan, heroin (Lockridge, 1990; Li et al., 2005). Plasma BChE also hydrolyzes prodrugs such as isosorbide diaspirinate, bambuterol (Li et al., 2005), and ISDA, a new aspirin prodrug (Moriarty et al., 2008). 

Plasma BChE is the major detoxifying enzyme of cocaine in humans (Inaba et al., 1978) and has been demonstrated to efficiently protect animals against cocaine toxicity (Hoffman et al., 1996; Lynch et al., 1997). However, BChE slowly hydrolyzes (-)cocaine with kcat/Km about 0.28 µM^-1^.min^-1^, so that under physiological conditions, a large part of the administered dose of cocaine reaches biological targets and triggers toxic effects. Mutagenesis efforts pursued over the last decade have dramatically enhanced the cocaine hydrolase activity of human BChE. A first mutation, A328Y, enhanced the catalytic efficiency 4-fold (Xie et al., 1999). Molecular dynamics simulation and computer-based ligand docking led to the A328W/Y332A double mutant that displays a higher kcat/Km=8.5 µM^-1^.min^-1^ (Sun et al., 2002). Further mutations using random mutagenesis raised kcat/Km. The simulation of activation transition state approach was also successfully applied to the design of new BChE mutants. Using the three-dimensional structure of human BChE (Nicolet et al., 2003), molecular dynamic simulations of the deacylation transition state led to the design of highly active mutants against (-)cocaine. This was achieved by combining four mutations, A199S/S287G/A328W/Y332G, and yielded an enzyme with a catalytic efficiency 456-fold greater than that of wild-type BuChE (Pan et al., 2005; Zheng and Zhan, 2008). The efficient quadruple mutant was fused to human albumin to improve its pharmacokinetics properties (t1/2=8h in plasma) without altering its catalytic efficiency (Brimijoin et al., 2008). At 10 mg/kg, the fusion enzyme stopped the symptoms of cocaine intoxication in rats at a lethal dose (100 mg/kg, i.p.). Interestingly, it also blocked cocaine-induced reinstatement of drug-seeking in rats that had previously self-administered cocaine. More recently, introduction of 5 mutations, A199S/F227A/S287G/A328W/Y332G, led to a more active enzyme (Zheng et al., 2008). Therefore, the fusion enzyme and new mutants are promising for an efficient therapeutic approach to cocaine overdose rescue and the treatment of addiction. Immunopharmacotherapy for cocaine addition can be an alternative strategy. Monoclonal antibodies catalyzing the hydrolysis of (-)cocaine have been made (Landry et al., 1993; Larsen et al., 2004; McKenzie et al., 2007). However, the kinetic parameters of catalytic antibodies have to be improved. Computational (transition state simulations, free energy barrier shift calculations) and mutagenesis approaches are expected to lead to more efficient biocatalysts (Pan et al., 2008).

## 3. Detoxification of organophosphorus compounds

**Fig. 2.**
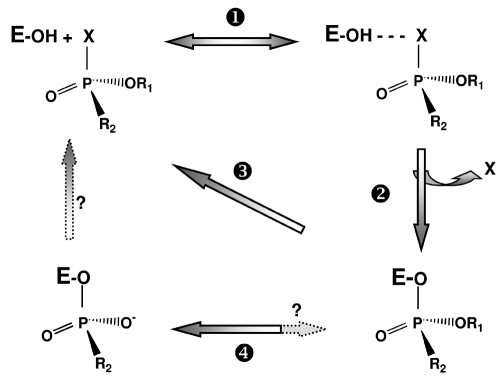
Inhibition of cholinesterases by OPs and reactivation of phosphylated enzymes After formation of reversible complex between ChE and OP (step 1), the active serine (esteratic site, E-OH) is phosphylated and there is release of OP leaving group X (step 2). Phosphylated ChEs can be reactivated by nucleophilic agents, such as oximes (Pralidoxime, MMB4, Obidoxime, HI-6, etc) used as antidotes in emergency treatment of OP poisoning (Lundy et al., 2006; Worek et al., 2007) (reaction 3); water is a too weak nucleophile for fast spontaneous reactivation of phosphylated ChEs. Phosphyl-ChE conjugates may undergo a dealkylation ('aging') (Shafferman et al., 1996; Viragh et al., 1997; Masson et al., 1999; Li et al., 2007; Carletti et al., 2008), resulting in irreversibly inactivated ("aged") enzyme (step 4). The dealkylation reaction can be very fast (t1/2 = 3 min at 37°C for human AChE phosphonylated by soman). At the moment, drug-mediated reactivation of aged ChE is not possible.

Organophosphates are widely used as pesticides. Some OPs are drugs, others are potent chemical warfare agents. OPs are irreversible inhibitors of cholinesterases (ChEs): acetylcholinesterase (AChE; EC 3.1.1.7) and butyrylcholinesterase (Fig. 2). AChE plays a major role in the cholinergic system terminating the action of acetylcholine. Thus, synaptic AChEs are the primary targets of OPs. Irreversible inhibition of AChE is the main cause of acute toxicity of OPs (Maxwell et al., 2006). No clear physiological function has been ascribed to BChE. In the nervous system and at neuromuscular junctions, BChE may surrogate AChE under certain conditions (Lockridge et al., 2009). However, BChE is of pharmacological and toxicological importance as shown in the previous section.

Endogenous enzymes are involved in natural defences against OP toxicity. The presence of OP detoxifying enzymes in skin contributes to reduce the amount of OP that penetrates into the body (Schallreuter et al., 2007). Certain secondary targets of OPs found in various tissues are detoxifying enzymes, and they certainly play a role in the natural defences against OPs (Wang et al., 1998; Nomura et al., 2005, 2008). Natural blood bioscavengers significantly contribute to reduce the amount of OP molecules reaching physiological targets. BChE is the most important stoichiometric OP scavenger in human plasma; its concentration is about 50 nM and its apparent second-order rate constant with OPs is ≈107 M^-1^ min^-1^. Human plasma PON1 displays activities categorized as "accidental" or "promiscuous" (Costa and Furlong, 2002; Mackness et al., 2008). Albeit its primary function is likely to be a lipophilic lactonase (Khersonsky and Tawfik 2005) involved in protection against atherosclerosis (Shih et al., 1998; Watson et al., 1995), it hydrolyzes numerous OPs. It has been shown that animals in which the plasma concentration in PON1 is high are relatively resistant to OPs (Kaliste-Korhonen et al. 1996). Conversely, knockout mice for PON1 are very sensitive to OPs (Shih et al., 1998). Albumin displays a low esterase activity and slowly reacts with carbamyl- and phosphoryl-esters. However, its concentration in the blood and lymph is high (≈0.6 mM), and it appears to play a significant role in the detoxification of carbaryl (Sogorb et al., 2007). Plasma albumin could also play a role in detoxification of OPs (Tarhoni et al., 2007; Li et al., 2008), even at toxicologically relevant concentrations (Sogorb et al., 2008). 

OPs are also neutralized by tissues (liver) carboxylesterases (CaE; EC 3.1.1.1) and oxidized by oxidases such as cytochrome P450s, glutathione S-transferases, laccases, and peroxidases. Glutathione S-transferases (GST; EC 2.5.1.18) are 20-30kDa enzymes that catalyze glutathione conjugation (nucleophilic attack of the thiol group) to electrophilic substrates. These enzymes are involved in the cellular detoxification processes of endogenous compounds and of numerous xenobiotics, and their role in the resistance of insects to insecticides is well established. OP detoxification by GSTs results from a regioselective dealkylation of the alkyl or aryl side chain (Maturano et al., 1997). There is evidence that GSTs contribute to OP detoxification in humans (Fujioka and Casida, 2007).

## 4. Stoichiometric bioscavengers against OPs

Administration of human plasma has been used for treating OP poisoning. The effects of fresh frozen plasma on cholinesterase levels and outcomes in patients with OP-poisoning were evaluated (Güven et al. 2004). Results suggest that plasma therapy may be an effective alternative or adjunctive treatment method. Plasma BChE and possibly other abundant OP scavenging proteins in plasma (albumin and PON1) may have contributed to this result.

Research on stoichiometric bioscavengers mostly focused on enzymes that specifically react with OPs; i.e. cholinesterases (Wolfe et al., 1987) and carboxylesterases (Redindo and Potter, 2005; Fleming et al., 2007). Prophylactic injection of enzymes capable of inactivating OP quickly would allow first responders; i.e firemen, explosive ordnance disposal technicians, and medical personnels to work safely in a contaminated environment. Intravenous or intramuscular administration of bioscavengers to chemical casualties is expected to greatly improve the efficacy of implemented pharmacological countermeasures (Ashani et al., 1998; Saxena et al., 2006). However, enzymatic stoichiometric neutralization of OP needs the administration of a huge amount of a costly bioscavenger, e.g., about 3 mg/kg of highly purified plasma BChE for challenging several LD_50_ of OP (Ashani and Pistinner, 2004). Large-scale production of enzymes under GMP conditions at a reasonable cost has been the subject of intense research in North America. 

Two industrial GMP processes exist for mass production of human BChE. The first one is purification of the natural enzyme from the Cohn Fraction IV of human plasma. One liter of plasma provides less than one milligram of GMP BChE. This process has been developped by Baxter Healthcare Corporation in the USA (www.baxter.com). Highly purified human plasma BChE was granted the status of Investigational New Drug by the Food and Drug Administration in 2006 for protection against nerve agents in the USA (Lenz et al., 2007; Saxena et al., 2007). Phase I of clinical trials on volunteers will be completed in Spring 2009, so that enzyme could be marketed soon. The second process has been developped by Nexia in Canada (www.nexiabiotech.com). It uses the recombinant human enzyme produced in the milk of trangenic goats. Several grams of enzyme can be secreted in one liter of milk. This recombinant enzyme has been named ProtexiaTM. Since 2005, the firm Pharmatheme in Maryland, USA (www.pharmathene.com), has been developping ProtexiaTM, PEGylated derivatives of the recombinant enzyme (Huang et al., 2007) and fusion proteins (Huang et al., 2008).

Secondary targets of OPs and other enzymes interacting with OPs are potential stoichiometric bioscavengers. In particular, owing to the high number of amino acid residues in albumin that covalently bind OP molecules (5 tyrosines and 2 serines) (Ding et al., 2008), the reactivity of tyrosine residues can be enhanced upon specific modification, e.g., nitration that causes a decrease in the pKa of tyrosine by several orders of magnitude (Masson et al., unpublished results). Modified human albumin could lead to a new generation of stoichiometric bioscavengers. Finally, low molecular stoichiometric scavengers could be an economic alternative to enzyme-based stoichiometric scavengers. Several serine- and tyrosine-containing hexapeptides from a random library of peptides have been selected, because they react with a fluorescent analogue of sarin (Landry and Deng, 2006; Deng et al., 2008).

## 5. Catalytic bioscavengers against OPs

As said, the detoxification of OPs involves the hydrolysis of the phosphoester bond by organophosphorus acid anhydride hydrolases (OPAH), also called phosphotriesterases (PTE), or oxidation to less toxic compounds by degrading their alkyl/aryl chain. Though works on catalytic antibodies (Vayron et al., 2000; Jovic et al. 2005; Reshetnyak et al., 2007) have made some progress, the re-design and engineering of enzymes capable of degrading OPs is the most promising research field. These enzymes could be used as catalytic bioscavengers for prophylaxis and treatment of OP poisoning, for topical protection (Fisher et al., 2005), and for the decontamination of skin, mucosa and open wounds (Lejeune and Russell, 1999; Gill and Ballesteros, 2000). Immobilized OPAH in bioreactors can be used for the decontamination of water (Simo et al., 2008), as well as genetically engineered bacteria producing OPAH can be introduced in water effluents of decontamination units for the purification of contaminated water before recycling or washing up in the environment (Chen and Mulchandani, 1998).

## 5.1. Requirement for an enzyme to be an efficient catalytic bioscavenger against OPs

There are several general requirements for the use of enzyme-degrading OPs as medical countermeasures against OP poisoning. Enzymes must have a wide spectra of activity, and ideally, enantioselectivity for toxic stereoisomers. Their mass production under GMP conditions must be realizable at a reasonable cost. Long-term storage without activity loss must be possible under field conditions. Conformational stability can be optimized by chemical modification or the addition of stabilizers. 

Other requirements depend on the way of administration, the delivery system or galenic formulation of enzymes. For parenteral administration, it must be remembered that the toxicant concentration in blood, [OP], even in the most severe cases of poisoning, is always very low, well below the Km of the enzyme for OP substrates. So, hydrolysis of OP in blood is pseudo first-order (Masson et al., 1998): v = kcat/Km •[E].[OP]. The product of the bimolecular rate constant (kcat/Km) and enzyme active site concentration ([E]) is the pseudo first-order rate constant. Thus, the higher the catalytic efficiency (kcat/Km), the lower the dose of enzyme to be administered for cleaning the blood of toxic molecules in a very short time (t). The catalytic efficiency of enzymes can be increased by several orders of magnitude by mutagenesis or chemical engineering (Griffiths and Tawfik, 2003; Hill et al., 2003). The enzyme concentration in blood needed to drop [OP] to a non-toxic concentration in time, t, is [E] = X/(kcat/Km). X is the factor by which [OP] is reduced (X= Ln[OP]_0_/[OP]_t_) (Masson et al., 2008). Therefore, the second constraint is to maintain the bioscavenger concentration, [E], in the blood as high as possible for a long time. [E] is controlled by the enzyme pharmacokinetics and/or the frequency of injections. Bioavailability and the biological stability of injected stoichiometric or catalytic bioscavengers are important issues. Increasing the size of the enzyme by polymerization, conjugation to albumin, reducing the microheterogeneity of glycans, and chemical modifications of solvent-exposed surfaces improve the biological life of injected bioscavengers. Fast clearance of glycoproteins is often due to glycosylation defects. These can be corrected by chemical modifications. Pharmacokinetic studies of glycoproteins injected to animals showed that the enzyme clearance depends on sialylation of glycans. Rapid elimination of asialoglycoproteins from the blood stream is due to their capture by galactose receptors located on the surface of hepatocytes. Galactose is the residue that precedes sialic acid at the terminus of complex glycans. Pharmacokinetic studies with natural and recombinant ChEs confirmed the importance of sialic acid residues ending glycans (Kronman et al., 1995; Saxena et al., 1998; Cohen et al., 2007; Kronman et al., 2007). It was found that the half-life, t1/2, is inversely proportional to the number of unoccupied attachment sites of sialic acid (Kronman et al., 2000). To increase t1/2 of administered recombinant ChEs, all galactosyl residues have to be sialylated. Full sialylation of recombinant enzymes can be achieved by the selection of an appropriate expression system (Chitlaru et al., 1998) capable of synthesizing glycans similar to natural human glycoprotein glycans and adding inhibitors of sialidase in the cell culture medium. Co-expression of human AChE and sialyltransferase in HEK 293 cells was found to produce fully sialylated recombinant enzyme (Kronman et al., 2000). Alternatively, in vitro polysialylation of purified enzymes is possible with a sialyltransferase or by using a chemical method (Gregoriadis et al., 1999). PEGylation was also proved to be an effective chemical modification for increasing the circulatory half-life of administered recombinant ChEs (Cohen et al., 2006, 2007; Huang et al., 2007; Kronman et al. 2007; Mazor et al., 2008; Chilukuri et al., 2008). Recently, a 150 kDa recombinant fusion protein human albumin-human BChE showed substantially improved pharmacokinetics when administered to juvenile pigs, t1/2 ≈ 32h against ≈ 3h for recombinant 70%-tetrameric BChE (Huang et al., 2008).

Immunotolerance of injected enzymes is a major issue. For instance, bacterial enzymes are not suitable for use in humans, but conjugation to dextran, PEG or inclusion in nanocontainers can reduce antigenicity and slow down clearance.

Enzymes in skin and eye lotions, immobilized in foams and on tissues for skin and eye decontamination (Gordon et al., 2003; Simo et al., 2008), or in topical skin protectants (Braue et al., 2002), act under conditions where local [OP] can be very high. In this case, enzyme reaction order in [OP] tends to zero, so that the reaction rate is close to maximum velocity, kcat.[E]. Thus, under these conditions, efficiency depends on the concentration and catalytic constant of the enzyme. Therefore, for external use, enzymes have to display high molecular catalytic activity and be highly concentrated. Co-immobilization of different enzymes could be an easy way to extend the spectra of agents to be degraded. This should allow simultaneous detoxification of different agents. Indeed, exposure to multiple agents has to be considered. In particular, in asymmetric conflicts, eschatological and criminal terrorism acts, the most extreme scenario has to be anticipated. 

## 5.2. Potential enzymes 

Cholinesterases - OPs may be regarded as hemi-substrates of ChEs (Fig. 2). When the enzyme reacts with carboxyl-esters, it is transiently acylated, the acyl group being rapidly displaced by a water molecule. On the contrary, with phosphyl-esters, the stereochemistry of the phosphyl-enzyme intermediate restricts the accessibility of water to the phosphorus atom. Thus, the spontaneous hydrolysis of the phosphylated enzyme is very slow or even impossible. Jarv postulated that the introduction of a second nucleophile pole in the active center could activate a water molecule. This water molecule could subsequently attack the phosphorus atom on the back face, leading to breakage of the P-serine bond (Jarv, 1989). The resolution of the three-dimensional structure of Topedo californica AChE (Sussman et al., 1991) opened the way to the rational re-design of ChEs. Thus, the possibility to convert ChEs into OP hydrolases (OPHs) was hypothesized. Human BChE was chosen because its active center is larger (500 Å3) than that of AChE (300 Å3) and it is less stereospecific. Molecular modeling based on the structure of Torpedo AChE was used for making mutants of human BChE. The second nucleophile pole was created in the oxyanion hole of the active center where a glycine residue was replaced by a histidine. This first mutant, G117H, was capable of hydrolyzing paraoxon, sarin, echothiophate and VX (Millard et al., 1995; Lockridge et al., 1997). However, it was irreversibly inhibited by soman, because "aging" of the conjugate was faster than dephosphonylation. The mechanism of aging, i.e. dealkylation of an akyl chain on the phosphorus atom, is almost completely elucidated (cf. Fig. 2). This reaction involves a carbocationic transient that is stabilized by active site residues E197 and W82 and water molecules (Shafferman et al., 1996; Viragh et al., 1997; Masson et al., 1999; Nachon et al., 2005; Li et al. 2007). Mutation of E197 into D, Q or G brought doun the rate of aging. As expected, the double mutant G117H/E197Q was capable of hydrolyzing soman (Millard et al., 1999). However, the catalytic activity of the double mutant was too slow to be of pharmacological interest. 

The discovery of a fly (Lucilia cuprina) resistant to OPs because it carries a mutated carboxylesterase (CaE) at a position homologous to G117, i.e G137D, stimulated research on G117H-based mutants of BChE. Though the OPAH activity of the G137D mutant is low, it is balanced by the abundance of the enzyme in insect organs (Newcomb et al., 1997). Mice knockout for AChE and carrying the G117H mutant of human BChE were found to be less sensitive to OP than wild-type mice (Wang et al., 2004). Though these mice express the G117H mutant in all organs, unlike the resistance of Lucilia cuprina, their resistance to OP cannot be explained by the OP hydrolysis that is too slow, but rather by the hydrolysis of excess acetylcholine that accumulates in cholinergic synapses.

More than 60 double or triple mutants of human BChE with mutated G117 (Schopfer et al., 2004) and mutants of human AChE and Bungarus fasciatus AChE were made using the same rationale (Poyot et al., 2006). For a review of these works, see Masson et al., (2008). None of the mutated ChEs was more active than the G117H mutant, and we provided evidence that mutations at position G117 cause dislocation and loss of functionality of the oxyanion hole (Masson et al., 2007). 

However, computer-assisted design of new OPH mutants of ChEs is conceivable using a new approach called "intelligen" directed mutagenesis design based on the simulation of transition states. As mentioned, this approach was successfully applied for making BChE mutants that hydrolyze (-)cocaine at a high rate. Simulation of dephosphylation transition states is expected to indicate how to optimize interactions favouring productive crossing of the dephosphylation energetic barrier. Directed evolution of ChEs could be an alternative to computer-based methods. However, functional expression of ChEs is difficult in yeast (Durova et al., unpublished results) and has failed in bacteria so far. 

Certain ChE mutants sensitive to OPs not susceptible to aging are fully reactivatable by oximes. Thus, ChE mutants associated with oxime reactivators act as pseudo-catalysts in displacing the OP moiety bound to the enzyme. These enzyme-reactivator-coupled systems could lead to a new family of pseudo-catalytic bioscavengers (Taylor et al., 2007; Kovarik et al., 2007, 2008; Mazor et al., 2008). 

Phosphotriesterases - Enzymes that catalyse the hydrolysis of phosphoester bonds in OPs are ubiquitous, e.g. a prolidase named organophosphorus acid anhydrolase (OPAA) was identified in a strain of Alteromonas (Cheng et al., 1999), a diisopropylfluorophosphatase (DFPase) is abundant in the squid Loligo vulgaris, and paraoxonase-1 (PON1) is present in human plasma. Bacterial enzymes called phosphotriesterases (PTE; EC 3.1.8.1), or sometimes organophosphorus hydrolases (OPH), organo-phosphate-degrading enzymes (OpdA), show preference for OP compounds with P-O or P-S bonds. PTE are members of the amidohydrolases superfamily (Seibert and Raushel 2005).

Bacterial phosphotriesterases - Phosphotriesterases (PTE; EC 3.1.8.1) are encoded by the organophosphate degradation (opd) gene found in Pseudomonas diminuta, Flavobacterium sp., and Agrobacterium radiobacter. Genes similar to opd were also found in Archaea (Merone et al., 2005). Pseudomonas diminuta PTE is a 72 kDa dimeric bimetallic enzyme with Zn^2+^ involved in catalysis (Carletti et al., in press). Substitution of Zn^2+^ ions in the active site with Mn, Co, Ni, or Cd ions results in the almost full retention of catalytic activity. Following the first determination of the three-dimensional structure of P. diminuta PTE (Benning et al., 1994), a series of crystal structures, kinetic, and spectroscopic experiments were described. PTE catalysis proceeds via a SN2 mechanism with the formation of a pentacoordinated transition state. The mechanism of the nucleophilic attack and enzyme regeneration are largely debated, and the functional roles of divalent metal cations and amino acids in the active centre are not yet fully understood (Aubert et al., 2004; Samples et al., 2007; Chen et al., 2007; Wong and Gao 2007; Jackson et al., 2008). Recently, the structure of SsoPox (a hyperthermopholic PTE from the archeon Sulfolobus solfataricus) provided new information that led to a refined mechanism (Elias et al. 2008; Del Vecchio et al., 2009). No natural substrate of PTE has yet been identified (Ghanem and Raushel, 2005) and PTE is thought to have evolved from lactonase (Afriat et al., 2006), the PTE activity being considered as a promiscuous activity (Elias et al., 2008). Whereas the catalytic efficiency of PTE for paraoxon, the best substrate identified so far, is approaching the diffusion-controlled limit, it is slow against OP nerve agents (Table 1). Meanwhile, directed evolution of PTE showed that only 3 amino acid changes dramatically enhanced the catalytic efficiency for an analogue of soman by ~3 orders of magnitude (Hill et al., 2003). 

**Table 1:** Catalytic efficiency (k_cat_/K_m_, M^-1^ min^-1^) of different natural and engineered OP hydrolases towards different OPs

Source of enzyme	Paraoxon	DFP	Tabun	Sarin	Soman	GF	Echothiophate	VX
Human PON1 Q192	6.8 x 10^5^^[a]^	4 x 10^4^^[b]^		9.1 x 10^5^^[c]^	2.8 x 10^6^^[c]^			+ ^[d]^
Human PON1 R192	2.4 x 10^6^^[a]^			7 x 10^4^^[c]^	2.1 x 10^6^^[c]^			+ ^[d]^
Human rPON1 in 293T					6.2 x 10^5^-4.1 x 10^6^^[e]^			
Mammalian rPON1 G3C9	7.2 x 10^5^^[f]^							
Mammalian rPON1 V346A					8.7 x 10^4^^[g]^	3.6 x 10^5^^[g]^		
Human BChE G117H	5.7 x 10^3^^[h]^	5.2 x 10^3^^[h]^		1.6 x 10^2^^[i]^	-		1 x 10^4^^[h]^	1.5 x 10^3^^[i]^
Blowfly CaE G117D	2 x 10^5^^[j]^						
*B. fasciatus* AChE HQT	64 ^[h]^	7.6 x 10^2^^[h]^					24 ^[h]^	
*Loligo vulgaris *DFPase		7.8 x 10^7^^[k]^		2.4 x 10^6^^[k]^	2.4 x 10^6^^[k]^			0 ^[k]^
*P. diminuta* OPAH	2 x 10^9^^[l]^	5.8 x 10^8^^[m]^		4.8 x 10^6^^[n]^	6 x 10^5^^[n]^	5 x 10^3^^[o]^		4 x 10^4^^[p]^
*Alteromonas sp.* JD6.5 OPAA		4.6 x 10^7^^[q]^			14.6 ^[r]^			
*Alteromonas sp.* JD6.5 cloned				5.8 x 10^6^^[r]^	1 x 10^7^^[r]^	6.2 x 10^7^^[r]^		
Alteromonas undina			21.8 ^[s]^	30.4 ^[s]^	1.6 x 10^2^^[s]^	1.3 x 10^2^^[s]^		
NG108-15 hybrid cells					2.5 x 10^3^^[t]^			

Catalytic efficiency (kcat/Km, M^-1^ min^-1^) of different natural and engineered OP hydrolases towards different OPs [a] Smollen et al., 1991; [b] Masson et al., 1998; [c] Davis et al., 1996; [d] C.A. Broomfield, unpublished result; [e] Yeung et al., 2008, with the four soman stereoisomers; [f] Harel et al., 2004; [g] Amitai et al., 2006; [h] Poyot et al., 2006; [i] Lockridge et al., 1997; [j] Newcomb et al., 1997; [k] Hartlieb and Ruterjans 2001; [l] Kuo et al., 1997; [m] Lai et al., 1995; [n] Dumas et al., 1990; [o] Hoskin et al., 1995; [p] Rastogi et al., 1997; [q] Cheng et al., 1999; [r] Cheng et al., 1994; [s] DeFranck et al., 1993; [t] Ray et al., 1988.

Numerous studies have highlighted the potential of PTE for decontamination, skin protection, and biosensor detection of OP (Lejeune and Russell, 1999; Gill and Ballesteros, 2000; Létant et al., 2005; Ghanem and Raushel, 2005; Karnati et al., 2007). Administration of PTE before or after OP exposure was shown to improve pre-treatment and current treatment of OP poisoning (Doctor and Saxena, 2005). OpdA was shown to improve survival after poisoning by highly toxic OP pesticides (Bird et al., 2008). To prevent abnormally fast pharmacokinetics and/or an immunological response of injected bacterial enzymes, PTE can be PEGylated (Jun et al., 2007, 2008) or encapsulated. First attempts at using PTE encapsulated within sterically stabilized liposomes were promising, providing protection to rats from multiple LD_50_ of OP pesticides (Petrikovics et al., 2004). Alternatively, blood detoxification could be achieved by extracorporeal circulation through a hollow fiber cartridge coated with immobilized PTE (Masson et al., unpublished results). 

PTEs possibly could also be used for skin protection as active components of topical skin protectants (TSPs) or covalently coupled to the cornified layer of epidermis (Parsa and Green, 2001). Thermostable PTEs from thermophilic bacteria (Merone et al., 2005; Elias et al., 2008; Del Vecchio et al., 2009) or mutated/evolved highly stable enzymes from mesophilic bacteria are promising for topical protection and decontamination. PTE was also entrapped in additives for latex coating of biodefensive surfaces. Such PTE-based additives for paints and coatings were shown to retain catalytic parameters and the stability of the enzyme (McDaniel et al., 2006). For the environment's decontamination and remediation, an alternative approach, phytodegradation by transgenic plants (e.g. tobacco) expressing a bacterial PTE, has been considered as a potentially low-cost, safe, and effective method (Wang et al., 2008).

**Fig. 3.**
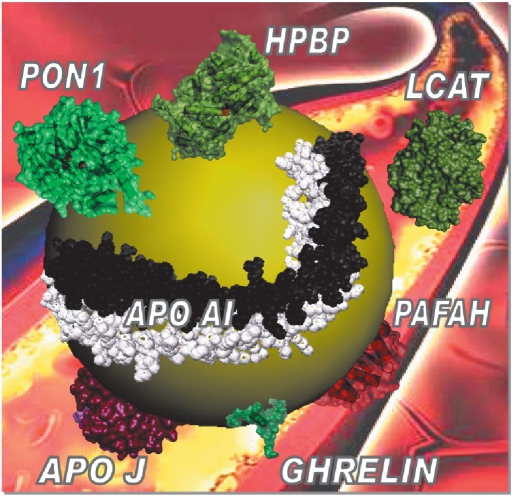
Scaled schematic representation for a PON1-containing HDL particle HDL is ~ 10 nm-diameter sphere with a non-polar core of cholesteryl-esters and triglycerides encapsulated in a monolayer of amphipathic α-helical apolipoproteins and phospholipids. Among multiple HDL-associated proteins involved in lipid metabolism, complement regulation, acute-phase response and proteinase inhibition (Vaisar et al., 2007), some of them, durably or transiently associated to PON1-containing HDL and described as having a propensity to contaminate purified PON1 fractions, are shown.

Human paraoxonase-1- PON1 is a 45 kDa glycosylated calcium-dependent enzyme expressed mainly in the liver and exclusively bound to high-density lipoproteins (HDL), in association with other apolipoproteins (Fig. 3). PON1 shows a genetic polymorphism; the most prominent determines the Q192R allozyme that has a substantial impact on PON1 activity with arylesters and OPs (Smolen et al., 1991) (Table 1). Chemical modification and site-directed mutagenesis studies have identified essential amino acid residues for the activities of PON1 (Josse et al., 1999; Yeung et al., 2004; Khersonsky and Tawfik 2006; Amitai et al., 2007; Tavori et al., 2008; Hu et al., 2009).

Attempts at solving the three-dimensional structure of human PON1 have failed so far. However, molecular modeling (Fokine et al., 2003; Yeung et al., 2004) and the crystal structure of a hybrid mammalian recombinant PON1 variant obtained by directed evolution (rPON1) (Harel et al., 2004) showed that PON1 is a six-bladed β-propeller protein similar to Loligo vulgaris DFPase (Katsemi et al., 2005). The catalytic mechanism of DFPase was recently described (Blum et al., 2006). Confirmation of a similar mechanism for human PON1 activity would considerably help to design more active PON1 mutants. The active site lid of PON1 is in close contact proximity to the area presumed to mediate binding of PON1 to HDL. It has been suggested that the hydrophobic N-terminus of PON1 mediates its anchoring to HDL, but the precise mode of binding of PON1 to HDL, as well as of other HDL-associated proteins, is still unknown.

As a naturally occurring plasma enzyme, human PON1 is the most promising catalytic bioscavenger candidate for pre-treatment and therapy of OP poisoning (La Du, 1996; Rochu et al., 2007a). Thus, PON1 is the focus of intensive research to improve its efficacy and functionalization. To provide a valuable medical countermeasure against intoxication by nerve agents, the catalytic efficiency of PON1 has to be enhanced by only 1 or 2 orders of magnitude. Mutants of rPON1 obtained by directed evolution and exhibiting enhanced OP-hydrolase activity (Amitai et al., 2006) suggest this goal can be reasonably achieved soon. However, the instability of these mutants could impinge on their biotechnological development. Plasma PON1 works in a complex and dynamic milieu, HDL particles that include durably or transiently up to 90 associated proteins (Vaisar et al., 2007). Thereby, PON1 requires association to apolipoprotein partners to retain its stable active conformation (James and Deakin, 2004; Gaidukov and Tawfik, 2005).

Human Phosphate Binding Protein (HPBP), an apolipoprotein that binds inorganic phosphate in blood was discovered recently. Its three-dimensional structure and complete amino acid sequence were solved (Morales et al., 2006; Diemer et al., 2008). The conditions to separate HPBP and PON1 in vitro indicate that HPBP is tightly associated with PON1 (Renault et al., 2006). Moreover, stabilization of the active form(s) of human PON1 by HPBP shows that HPBP acts as a functional chaperone (Rochu et al., 2007b, c; Cléry-Barraud et al., 2009).

We are attempting at co-crystallization of the PON1-HPBP complex. For this, the first step was to construct a hybrid gene for co-expression of HPBP and PON1 in E. coli. For this purpose, a HPBP gene was synthesized from its amino acid sequence. Co-expression, aimed to favour correct folding of active PON1 and stabilization of the active functional conformation of the enzyme, is expected to provide a crystallizable PON1-HPBP complex. Finally, diffractable crystals of the complex are assumed to provide a three-dimensional structure of natural human PON1. This crucial phase will be the first step of the staircase, leading to the development of stable human PON1 mutants with enhanced catalytic efficiency against OPs. The fact that functional expression of human PON1 seems to be feasible (Stevens et al. 2008) would simplify the biotechnology of PON1 and open new perspectives.

Other enzymes - Other hydrolases are involved in biodegradation of OPs, such as organophosphorus acid anhydrolases (OPAA; EC 3.1.8.2) and organophosphate-degrading agents (OpdA) or prolidases (EC 3.4.13.9). Prolidases were first isolated from halophilic bacteria (Alteromonas haloplanktis and A. sp. JD6.5). Prolidase from A. sp. JD6.5 is an OPAA that displays the highest known activity against soman (kcat = 3100 s^-1^), but it is inactive against VX (Cheng et al. 1999). OpdA was incorporated as the active ingredient of Landguard™ OP-A, a formulation developped for the treatment of water run-off, equipment rinsing and soil decontamination (Dawson et al., 2008). Whilst these applications are focussed on agricultural and remediation markets, modifications required for nerve agent detoxification will be relatively minor, as the enzyme can be applied in powder, liquid or matrix-bound forms. 

A prolidase was also isolated from human liver. This prolidase displays a high catalytic activity against soman and exhibits sequence homology with the Aleromonas haloplanktis prolidase (Wang et al., 1998). Human prolidase has been cloned and expressed in Sacharromyces cerevisiae and Pichia pastoris, and it appears to be one of the most interesting OP-degrading enzyme for protection against nerve agents (Wang et al., 2005; Wang et al., 2006). The newly discovered human cytosolic aminopeptidase (AMPP; EC 3.4.11.9) could also be a valuable catalytic bioscavenger against organophosphates and organophosphonates (Hsu et al., 2008).

Laccases (EC 1.10.3.2) are fungal phenol oxidoreductases that have been used for detoxification of numerous xenobiotics (Richardt and Blum, 2008). The laccases from Pleurotus ostreatus and Chaetomium thermophilium were found to rapidly degrade VX and VR in the presence of 2,2'-azinobis(3-ethylbenzthiazoline-6-sulfonate (ABTS) as the mediator (Amitai et al., 1998). We found that laccases from Trametes versicolor and Coriolopsis polyzona with ABTS display similar properties against V agents (Trovaslet et al., unpublished results). The heme-containing chloroperoxidase (EC 1.11.1.X) from Caldaromyces fumago, with peroxide as co-substrate, is another efficient VX-degrading enzyme (Amitai et al., 2003).

These enzymes are of interest for the destruction of chemical weapons stockpiles, soil remediation, the decontamination of materials, protective equipments, and water polluted by pesticides and nerve agents (Russel et al., 2003). In particular, phosphorothiolates such as VX are relatively resistant to PTE. Thus, oxidative cleavage of the P-S bond could be achieved by oxidases like laccases. These enzymes could be used associated with other OP-degrading enzymes for skin decontamination and topical skin protection. Though no work has been performed on the combined action of oxidases and hydrolases, oxidation of P-bonded alkyl/aryl chains by oxidases is expected to alter enantioselectivity of PTE for parent OPs, and therefore to improve the efficiency of catalytic bioscavengers.

## 6. Conclusions and future directions 

Enzymes that degrade OPs have been isolated from humans, different animal species, fungi and bacteria. Identification and isolation of new natural enzymes, in particular in insects resistant to OP pesticides and among secondary targets of OPs in humans is an active field of research. Also, potential extremozymes have been discovered in halophilic, thermophilic and piezophilic bacteria and other extreme environments (Merone et al., 2005; Feerer et al., 2007). All these enzymes can be purified from their natural sources. However, production of natural enzymes free of infectious agents under GMP is expensive. Thus, suitable biopharmaceuticals are mostly recombinant enzymes. These enzymes can be produced in prokaryotic expression systems (E. coli), eukaryotic expression systems (yeast, insect, mammalian cell cultures), transgenic animals (worm, rabbit, goat), transgenic plants (tomato, potato, tobacco), and also acellular biosynthetic systems. Current research's goal in protein engineering is to improve mass production of stable enzymes at low cost. 

Improvement of in vitro and in vivo catalytic properties of cocaine hydrolases and OPAH-degrading nerve agents and pesticides is the main issue. Improvement of thermodynamic stability for long-term storage in solution or in lyophilized forms, and in vivo operational stability, improvement of immuno-tolerance and bioavailability are other major goals. Molecular modeling and transition state simulations, site-directed mutagenesis and directed-evolution approaches in combination with chemical modifications and medium manipulations have been successfully used to improve the properties of selected enzymes (Bershtein and Tawfik, 2008). Lastly, pharmacokinetic, toxicokinetic and immunological studies on animal models, and then on volunteers will validate enzymes of interest. Multiple enzymes will be associated in active topical skin protectants and decontamination tools. Soon, catalytic bioscavengers are going to take a place among medical countermeasures for prophylaxis and treatment of acute OP poisoning and treatment of cocaine overdose.

In the future, gene therapy could be considered to challenge OPs. This strategy will offer the possibility of transitory production of humanized OP-degrading enzymes in the body. Promising results have already been obtained with human AChE and BChE (Li et al., 2006; Chilukuri et al. 2008) and human PON1 (Conwan et al., 2001; Fu et al., 2005; Bradshaw et al., 2005; Miyoshi et al., 2007; Guns et al., 2007; Zhang et al. 2008). Again, the use of mutated PON1 genes encoding for an enzyme with high OPH activity against pesticides and nerve agents appears to be the most promising approach. Several studies, using different gene-delivery vectors in mice, showed that the level of PON1 in plasma was increased. High PON1 levels slowed and even prevented the entry of OP into the brain and reduced atherosclerosis signs (Conwan et al., 2001; Fu et al., 2005; Bradshaw et al., 2005; Guns et al., 2008). Local delivery of the PON1 gene using the Sendai virus vector inhibited neonatal hyperplasia after arterial balloon-injury in rabbits fed a high-fat diet (Miyoshi et al., 2007). Transfer of the human PON1 Q gene in mice led to efficient expression of the enzyme that was capable of protecting the liver against oxidative stress (Zhang et al. 2008). Thus, enhanced expression of PON1 by gene therapy could be beneficial to the different functions of the enzyme. Meanwhile, the multiple and defectively identified PON1 activities make it apparent that strategy for repetitive administration of high concentration of PON1 in humans must be undertaken with caution. 
